# Supplementary figures and images for: Particle retracking algorithm capable of quantifying large, local matrix deformation for traction force microscopy
Source: PLoS One. 2022 Jun 22;17(6):e0268614. doi: 10.1371/journal.pone.0268614 (PMC9216574; doi:10.1371/journal.pone.0268614)

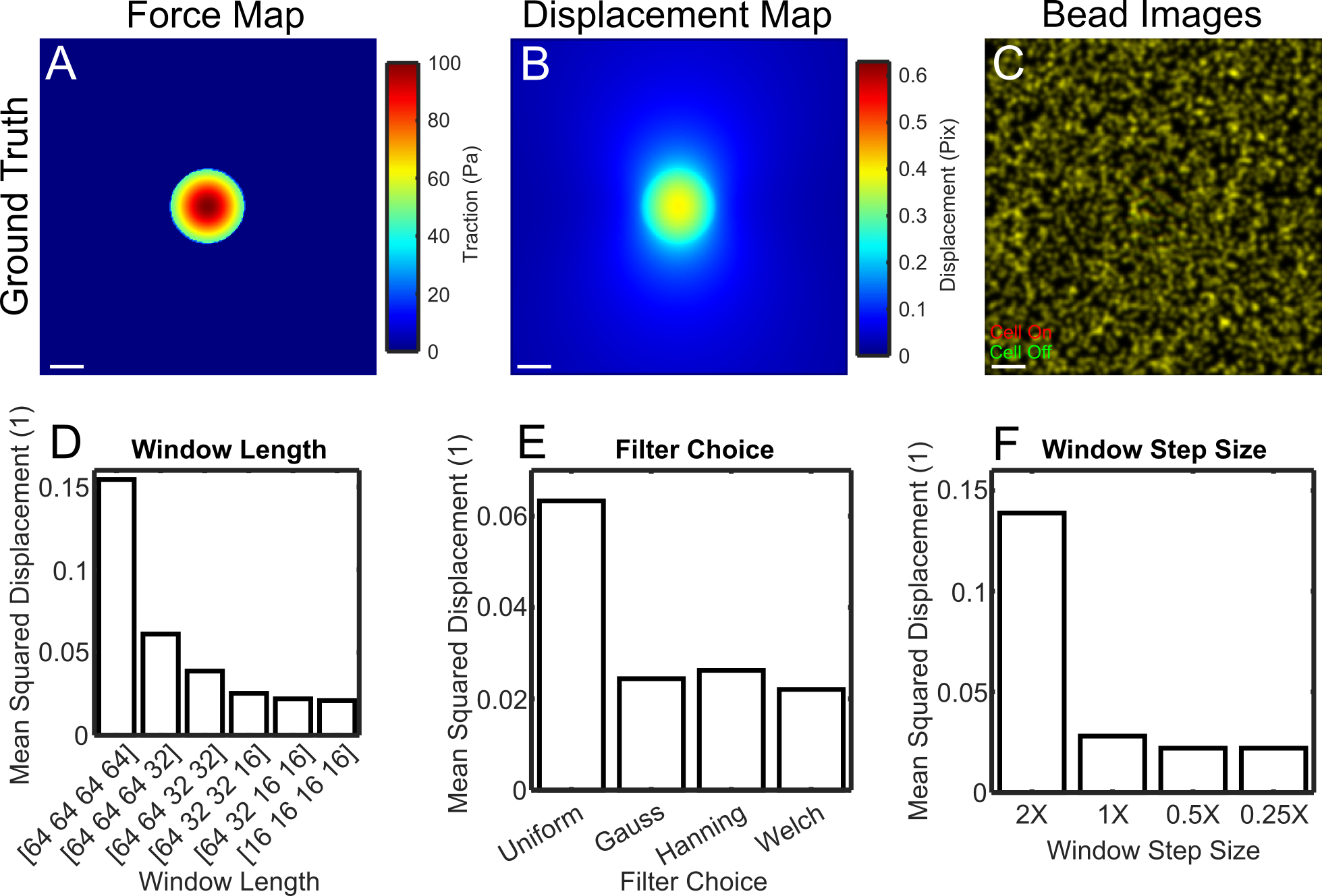

Supplement: S1 Fig — In this test, the template size, a kind of filter, and the grid spacing of PIV Suite, as a representative of PIV methods, was tested on the bead images containing intermediate displacement. (A) Ground-truth traction map showing intermediate level (~100 Pa) of traction in the center. All traction vectors (invisible) are directing downward, i.e., negative y-direction. (B) Ground-truth displacement magnitude map calculated using Boussinesq solution with E = 1kPa. (C) A color-merged image of a synthetic bead image (8000 beads on 512x512 pixel area) in a relaxed configuration (red) and another image of beads where displacements in A is applied (green). Scale bars in A-C: 1 μm assuming 108 nm/pixel. (D-F) Bar plots of the mean-squared-deviation (MSD) for the measured displacement fields by PIV Suite with different template window lengths (D), template filters (E) and grid spacing or window step size (F). Parameters not being tested were maintained as in Table 1. For panel F’s x-label, 1X means that the step size is the same length as template window length, i.e., 16 pixel in the case. These tests demonstrate that small-enough template window, step size and Welch filter produce the best tracking results. (TIF) [file pone.0268614.s001.tif]
